# On the Employment of Heat in the Treatment of Ulcers, Wounds after Amputation, and Other Great Surgical Operations, of Hysteria, Diseases of the Skin, &c.

**Published:** 1845-01

**Authors:** 


### ART. XVII.

*De l'emploi de la Chaleur dans le Traitement des Ulcères, des Plaies après les Amputations et les grandes Opérations chirurgicales, de l'Hysterie, des Maladies de la Peau, du Rheumatisme, &c.* Par M. LE DR. JULES GUYOT.—*Paris*, 1842.

*On the Employment of Heat in the Treatment of Ulcers, Wounds after Amputation, and other great Surgical Operations, of Hysteria, Diseases of the Skin, &c.* By M. LE DR. JULES GUYOT.—*Paris*, 1842. 8vo, pp. 264.

THIS work is divided into four parts. The first consists of pure theory, with deductions from that theory, and from observations anterior to those of the author; the second treats generally of the effects of natural and artificial heat, and of the means of applying the latter; the third part contains the results of the author's experience in the remedial application of heat at a certain temperature; the fourth part consists of an analysis and discussion of the facts previously adduced, with some general precepts and inductions.

The theoretical part need not detain us long. According to M. Guyot, the efficient cause of organization is *heat*, which, being at first derived from extraneous sources, afterwards becomes the most immediate and necessary result of organization; there exists, therefore, in all living beings, a vital principle, which is neither more nor less than the degree of heat appropriate to each species. M. Guyot considers the following as three absolute laws, subject to no exception:

"1. The proximate and determining cause of the formation of all beings organized for the purpose of living, is heat.

"2. No being so organized can live, unless it either maintain internally the same degree of heat which has produced its organization, or receive that heat from some extraneous influence.

"3. Affinity, organic contractility and sensibility are immediately dependent on the temperature proper to each living being, and the same is the case with the contractility and sensibility termed animal." (p. 36.)

We think our author would have done far better to have eschewed all theory, and gone at once into the practical part of his subject The truth is, even admitting that any benefit could arise from speculating on such a theme, M. Guyot is not precisely in a conditiou to do so with effect, being evidently by no means profound in organic chemistry—a most essential study to one who would enter such a field of inquiry, with the smallest prospect of success.

Waiving the question whether heat be or be not the vital principle, it is quite clear that temperature has immense influence on all the normal prosesses and developments of life, and it may hence be reasonably inferred that its influence is also very extensive on morbid and reparative progresses and developments. The latter consideration, though sufficiently obvious, has been much neglected, and the influence of temperature on the progresses of diseases, and the reparation of injuries has hitherto been but very partially investigated. The work of M. Guyot, however, though calculated to awaken attention generally to the remedial effects of temperature, regards the subject principally under a phase which is entirely new, and which may prove to be highly interesting and important. His main inquiry relates to the effects of *a particular temperature*, which is that established by nature as most favorable to the healthy processes of life, and which may thence be presumed to be favorable also to those efforts by which nature opposes morbid actions, and effects the reparation of parts injured by disease or violence. Our author deserves credit for having taken up this inquiry, as well as for the diligence with which he has pursued it; we have therefore much pleasure in presenting our readers with an abstract of his researches.

In the second part of his work M. Guyot treats of "natural and artificial incubation." Incubation he defines to be "the prolonged application of a certain heat adapted to favour the development of an organization capable of living." The natural internal heat of the human body, according to our author, is 36° cent. (96⅘° Fahr.)

"When man has arrived at his full development, he contains within himself, and at every point of his organization, a sufficient temperature. It would be contrary to his organization and his health that incubation should be prolonged beyond the limits imposed by the laws of nature. The excitement which he receives from an extraneous temperature different from his own, is indispensable to the activity of his functions; but it is this very excitement which becomes baneful, when the functions are altered or suspended. Every temperature different from their natural heat stimulates and irritates the organs, to which it is applied. If the functions of these organs he deranged, they will become so much the more so, in that the organs will have to sustain a more active contest with external impressions; and the more the external temperature differs from the natural heat of the body, the more will the body suffer violence and exhaustion; it is indifferent whether it have to contend with an extreme of heat or of cold, the phenomenon of irritation is nearly the same in both cases." (p. 49.)

Previously to inquiry into the best means of sustaining around the body a temperature of 36° cent., M. Guyot considers the question whether the contact of air with the living tissues, its penetration into organs, or its action upon wounds be attended with any mischievous effects. In 1831, M. Guyot published a memoir in the 'Annales de la Médecine Physiologique,' containing the results of his experiments on animals relative to the above question. His inferences from these, and other more recent experiments are as follows: Air, when devoid of any noxious impregnation, has, simply considered, no injurious action on living parts. It may, nevertheless, become injurious in two ways: 1. Mechanically, as by causing the lung to collapse in penetrating wounds of the chest; by destroying the cellular attachments of the viscera in wounds of the abdomen; and by distending and compressing organs in emphysema. 2. As the medium of an injurious temperature. Air, introduced into the serous and synovial cavities occasions inflammation by its coldness: at the temperature of 36° cent., the articular, cranial, thoracic, and abdominal cavities may be opened without danger of inflammation, in as far as the contact of the atmosphere is concerned.

M. Guyot adopts air as the vehicle of the "heat of incubation" because it unites all the physiological conditions appropriate to our organs, which are not fitted to sustain long submersion in water, or the continued action of humidity; neither can they support a water-heat at the degree of incubation; lastly, water, cataplasms, &c. penetrate and swell the epidermis and other parts, so that the bad effect of the humidity neutralizes, in some degree, the beneficial influence of the heat, although there are certain cases in which moisture may be advantageously combined with heat.

By incubation, then, M. Guyot understands a temperature of 36° cent. applied to the body by means of heated air. He distinguishes three species of incubation:

1. Local or circumscribed, as that adapted to amputations, wounds, ulcers, white swellings, &c. the action being confined to the part to which the remedy is immediately applied.

2. Diffuse, acting on a considerable portion of the body, to reestablish a function, or restore the equilibrium of the circulation or innervation, as in chlorosis, amenorrhea, phlegmasia dolens, extensive œdema, ascites, neuralgia, &c., and in affections which have no defined localty to which heat can be applied.

3. General incubation, which might be applied in rachitis, scrofula, premature birth, or defective development of children, &c.

Incubation is also divided into constant, or that which is continued throughout the case, and intermittent, or that which is suspended from time to time. Continued incubation ought to be maintained day and night without any interruption, except that occasioned by dressings and the proper disposition of parts. Intermittent incubation may be applied for six or eight hours in the day, or all day and every day; it may be applied only during the night, and every night; or lastly, it may often be necessary to prolong the incubation for several days, to discontinue it for the like period and then renew, and again discontinue it in an alternate manner.

The remainder of the second part of M. Guyot's work is occupied with a description of the apparatus which he employs for the application of heat. For this we must refer the reader to the book itself, where the text is illustrated by plates, the absence of which would render any attempt at description on our part unprofitable.

The third part is occupied with a copious detail of cases exhibiting the results of the treatment by incubation. The cases in which M. Guyot has tried his peculiar treatment are the following:

*Ulcers.* Of sixteen ulcers, occurring in the persons of fourteen patients, nine were completely and permanently cicatrized; five were reduced from very large to very small dimensions; and two resisted the treatment entirely. Some of the cases which were cured were of an inveterate kind, and one patient was a man aged 67 with an ulcer on the inner malleolus of twenty-five years standing.

*Wounds and diseases of the skin and cellular tissue.* The cases which come under this head are: 1, A large callous and fistulous sore, resulting from an abscess in front of the *tendo Achillis,* and which had resisted all treatment for nine months. Under the treatment of M. Guyot the wound was healed in fifteen days with the exception of the fistulous orifices, which, however, now discharged a healthy pus. On the twenty-fifth day the superior fistulæ had ceased to suppurate, and on the thirty-second they were cicatrized and all callosity had disappeared. In thirty-six days there were no remains of disease, except the two inferior fistulous orifices. At this period M. Guyot lost sight of the case. 2. An immense lacerated wound of the left arm which had been torn by machinery. It was healed in twenty-three days. 3. A wound of the heel resulting from an operation for the cure of a callous ulcer. The wound itself had assumed the form of an ulcer, and would not heal. By the use of incubation it was healed in eighteen days. 4. A case of phlegmonoid erysipelas terminating in gangrene, which had destroyed the integuments on the anterior part of the foot, and the anterior and inferior half of the leg, and left, after the detachment of the sloughs, a profusely suppurating surface. The symptomatic fever was severe and typhoid, and accompanied with watery diarrhea. Two days after the commencement of the treatment by incubation, the suppuration was diminished to one half, the wound was covered with granulations, and the fever had ceased. In thirty-nine days the wound being nearly healed, it was consigned to ordinary treatment. 5. Phlegmonoid erysipelas in consequence of division of the tendo achillis; treatment successful. 6. Eczema of the right leg, of several years standing, cured in twenty-three days, a year having elapsed without any return of the complaint. 7. Crushing of the foot with wound of the integuments, and intense pain which did not yield to leeches or poultices. Incubation completely relieved the pain in two hours, and in nine days the patient was able to walk. 8. Œdema of the arm, forearm, and hand, with unhealthy suppuration, remaining after an attack of phlegmonoid erysipelas which had commenced at the elbow. The cure was complete in eleven days. 9. Œdema supervening on fracture of the thigh, with effusion (of what is not stated) into the knee-joint; treatment successful. 10. Phlegmonoid erysipelas, occurring in a cook who had struck his hand against a calf's tooth in cleaving the head; treatment successful. 11. Crushing of the foot with sloughing; treatment successful.

*White swelling.* Of this disease M.Guyot records 6 cases which were subjected to his peculiar treatment. In 4 the knee was the part affected, in 1 the shoulder, and in 1 the wrist. Of the knee cases, the 1st was not cured; but the instance is not in point, for the temperature employed was 45° (113° F.) raised to 60°·1,(140° F.) and not, therefore, the temperature of incubation. In the second case incubation was followed up by blisters and cauteries, and a complete cure was obtained. The third case grew worse under the treatment by incubation, which was therefore abandoned. In the fourth case an inprovement was effected, but it was only temporary. The shoulder case was of doubtful character, being considered by some as white-swelling, by others as spina ventosa, and by others again as a separation of the epiphysis, or fracture of the neck of the humerus, which had been neglected. However this may have been, the patient, a boy 10 years of age, was pallid and exhausted with pain, suppuration, repeated leeching, and continued low diet. The disease had originated four months before in a fall on the affected arm, which was succeeded by pain in the joint and, after fifteen days, by considerable inflammatory swelling, which gradually assumed the chronic and serious character presented by the case when it first came under M. Guyot's care. At the commencement of his treatment, he determined to apply the heat for ten hours only every day. The limb was so disposed as to prevent motion in the diseased parts; steel pills were prescribed, and a meat diet enjoined. In sixteen days the tumour took on a better aspect; the suppuration was less copious and less sanious; the pains were gone, and the tenderness on pressure was much abated. As the progress of the case was considered slow, the heat was now applied uninterruptedly. In four days more, the tumour was reduced to a third of its former circumference; the suppuration was very thick, and scanty. In less than a month the cure appeared complete. Some months after, however, tumefaction reappeared and two fistulous canals reopened, but there was no constitutional disturbance, and by a renewed application of the heat the symptoms were subdued, and the patient finally recovered with the perfect use of his limb.

*Rheumatism.* Only two rheumatic cases are given, both of the chronic kind. The first was periostitis of the tibia, originating in a blow, and accompanied by pains which, M. Guyot says, were "very evidently of a rheumatic nature:" this is not so perfectly evident to us, but as we do not know what the nature of rheumatism is, and as we are wont to hear the name of "chronic rheumatism" applied to all sorts of pains from all sorts of causes, we will not stop to discuss the matter. The second case was rheumatism of the knee of some weeks standing. Both cases were cured under the use of incubation, the one in eight, the other in twelve days.

In his "résumé," M.Guyot has associated, under one head, "affections lymphatiques, tumeurs blanches, rhumatismes." But "affections lymphatiques" is a very vague expression, and as no particular instances are given, we have discarded the "affections" altogether; we have also separated white swellings and rheumatisms, as things having no natural affinity.

*Hysteria.* One case of this disease is recorded, which was combined with chlorosis, and was of that irregular kind so frequently met with in practice, simulating various maladies at different times. The symptoms at the period when the patient came under the treatment by incubation were, entire sleeplessness, continual palpitation of the heart, nausea, vomiting, acute pain in the right iliac region, epigastrium, hypogastrium, and kidneys; extreme weakness; fainting on assuming the erect posture, &c. Incubation was applied to the lower half of the body, and, in six hours, produced one of those marvellously sudden transitions from disease to health, which not unfrequently occur, in hysterical cases under every variety of treatment. In six hours, the patient, a young lady of 19, was free from all complaint. For nearly a month she was subjected to incubation for six hours every day, and no further nervous symptoms appeared, the appetite and strength returned, and a complete cure was obtained.

*Malignant or ill-conditioned sores.* Two cases of this nature are mentioned. One is that of an ulcer, the particular characters of which are not specified occurring on the right side of the nose in a Hungarian Count. It was much benefited by incubation after having resisted ordinary treatment, but was not healed when the patient departed for his own country. The other case is that of a lady who had a cancerous ulcer of the worst character on the right side of the nose. M. Guyot conceives that the incubation which was here applied was useful only as a preliminary measure; it modified, in a remarkable manner, the mammellated and tuberculated surface of the sore, contracted its limits, caused the redness and pain to disappear, and predisposed the tissues to cicatrization. The arsenical paste was then applied, and the cure became complete.

*Amputations.* M. Guyot gives the details of thirty-two cases in which the treatment by incubation was applied to the stump. after various amputations. Of these cases he gives reasons why eight should not be taken into the account,—the unfavorable state of the patient, or the inefficient application of the treatment, rendering them unfair criteria. Of the remaining twenty-four cases, the results were as follow. Thirteen amputations of the thigh,—eight cures, five deaths; eight of the leg, —five cures, three deaths; one of the fore-arm, cure; one of the great toe, cure; one of the ring-finger, cure.

When we compare this statement with the average mortality after amputations in the Parisian hospitals, a wonderful contrast certainly appears in favour of incubation. M. Guyot affirms that the recoveries in those hospitals after amputation of the thigh are less than one in six, and tells us that M. Dupuytren said "one cure in three is good; but two cures, that's magnificent." We are not enabled to state with accuracy the average of mortality in these cases in the London hospitals; but we venture to assert that the surgeons attached to them would feel anything but elated by the success here characterized as magnificent. In considering the causes of M. Guyot's comparative success, it should not be forgotten that he seems always to adopt union by the first intention when it is practicable. This proceeding, in fact, makes the stump perform "incubation" upon itself, and it may be doubted whether the application of an artificial temperature, as practised by M. Guyot, would have any appreciable effect. One thing is certain, namely, that union by the first intention is quite sufficient to account for the superior success of any treatment wherein it is adopted, over any other in which it is not; and it is well known that this beneficial practice, though much more frequent in France than it used to be, is still not nearly so general as in this country.

*Puerperal peritonitis.* One case, which appears to have presented the symptoms of this disease in a severe form was very successfully treated by "incubation" applied to the abdomen.

We believe we have now placed before our readers the most important contents of M. Guyot's book. He is a very immethodical writer; there is little classification of cases throughout his work, and the fourth part, called an "analysis and discussion of facts," by no means assists us in reducing the rest to order; so that we have been obliged to bring his principal results together as we best could, in the hope of omitting nothing that was of much consequence.

Some of our author's theoretical views, when he does not strain them too far, are sufficiently rational, while the practice which he advocates appears to have been successful in his own hands, and is therefore entitled to an impartial trial at the hands of others.

